# Economic evaluation of a hospital-initiated tobacco dependence treatment service

**DOI:** 10.1136/bmjopen-2025-107111

**Published:** 2025-12-05

**Authors:** John Robins, Gary Alltimes, Irem Patel, Ann McNeill, John Moxham, Stephanie Duckworth Porras, Andrew Stock, Arran Woodhouse, Deborah Robson

**Affiliations:** 1 Addictions Department, King’s College London Institute of Psychiatry Psychology & Neuroscience, London, England, UK; 2 NIHR ARC South London, London, England, UK; 3 King’s College Hospital NHS Foundation Trust, London, England, UK; 4 Respiratory Medicine, King’s College Hospital NHS Foundation Trust, London, England, UK; 5 King’s College London Institute of Psychiatry Psychology & Neuroscience, London, England, UK; 6 Integrated Respiratory Team, King’s College Hospital NHS Foundation Trust, London, England, UK

**Keywords:** Tobacco Use, Smoking Reduction, Inpatients, Health Care Costs, HEALTH ECONOMICS

## Abstract

**Abstract:**

**Objectives:**

The treatment of tobacco dependence in patients admitted to hospital is a priority for the National Health Service in England. We aimed to conduct an economic analysis of a pilot ‘opt-out’ tobacco dependence treatment intervention adapted from the Ottawa Model of Smoking Cessation.

**Design:**

Observational cost analysis of an inpatient tobacco dependence treatment intervention, and matched cohort study comparing readmission costs between patients who received the intervention and benchmarked equivalents who did not.

**Setting:**

11 acute inpatient wards in a major teaching hospital in London, England.

**Participants:**

673 patients who smoked, admitted between 1 July 2020 and 30 June 2021.

**Interventions:**

The intervention consisted of the systematic identification of smoking status, automatic referral to tobacco dependence advisors, provision of pharmacotherapy and behavioural support throughout the hospital stay and telephone support for 6 months after discharge.

**Primary and secondary outcome measures:**

The primary outcomes were cost-per-patient, cost-per-quit and incremental cost effectiveness ratio among patients who received the intervention. The secondary outcomes were patient-level readmission costs and bed-days from 6 months after discharge, compared between the intervention group and a group of matched benchmark patients who smoked but did not receive the intervention.

**Results:**

The total cost of the intervention was £178 105. On the basis of 104 patients who reported not smoking at 6 months, the cost-per-quit was £1712.55, equating to an estimated age-adjusted incremental cost per life year gained of £3325. Among 611 patients who were successfully matched to a benchmark cohort, readmissions for patients in the intervention group cost £492 k less than their benchmark equivalents over 21 months from 1 January 2021 to 30 September 2022 (£266 k vs £758 k), incurred 414 fewer bed days (303 vs 717) and readmitted at a lower rate (5% vs 11%). There were reduced readmission rates and costs among all patients who received the intervention compared with their benchmarked equivalents, regardless of smoking status at 6 months, except among those who opted out.

**Conclusions:**

A pilot ‘opt-out’ tobacco dependence treatment intervention implemented in an acute hospital setting in London demonstrated value for money through reduced readmission rates and costs among all patients who received it.

**STRENGTHS AND LIMITATIONS OF THIS STUDY**

- Our study uses a well-validated Patient-Level Information and Costing Systems tool to attribute accurate healthcare costs to patient activity; an assessment of the National Health Service (NHS) Trust’s costing tool’s data quality and adherence to NHS Costing Transformation Programme standards was scored at 98% and has been ranked in the top five nationally.
- The estimated costs do not include postdischarge costs such as local authority Stop Smoking Services, to which some patients were referred, nor do they include wider societal costs.
- Postdischarge smoking status was not biochemically verified; however, this reflects the practical realities of treatment initiated during hospital admission and has been accounted for in our sensitivity analysis.
- Our study was conducted in an urban setting during the COVID-19 pandemic, and so, results may not be applicable to different contexts.

## Background

 Tobacco smoking remains the modifiable mortality risk factor that accounts for more years of life lost than any other.^1^ It increases the risk of a multitude of diseases, especially lung cancer, chronic obstructive pulmonary disease and coronary heart disease (CHD), and is the largest avoidable cause of disability and social inequalities in health in the UK.^2^ In 2019/2020 there were an estimated 506 100 admissions to hospitals in England that were attributable to smoking, costing an estimated £850 m.^3^

Recently, there has been significant new investment within the National Health Service (NHS) in England that focuses on the treatment of tobacco dependency for patients admitted to acute medical care, to psychiatric hospitals and to maternity services.^4^ The NHS Long Term Plan committed to ensuring that all patients admitted to hospital who smoked would be offered NHS-funded tobacco dependency interventions by 2024. Recommended interventions included the Ottawa Model for Smoking Cessation (OMSC), an ‘opt-out’ model which incorporates the systematic identification of the smoking status of all admitted patients, followed by brief advice, personalised bedside counselling, timely nicotine replacement therapy (NRT) and/or pharmacotherapy and follow-up after discharge.^5^ Hospital-initiated smoking cessation interventions have been found to be effective in increasing quit attempts and quit success^5^,^10^ and reducing readmission rates and mortality.^5 11^

There is less evidence regarding the cost-effectiveness of hospital-initiated smoking cessation interventions, although where such research has been conducted, the results are favourable, despite differences in delivery of the intervention and the methods of evaluation.^12^ The majority of the current evidence comes from the USA, which uses a different model of healthcare funding to countries such as the UK, the latter being taxpayer-funded and free at the point of use.^13^ Many of the health economic analyses of tobacco dependence treatment also come from randomised controlled trials, which are often concerned with the cost effectiveness of a single element of an intervention compared with its closest alternative, for example, standard versus enhanced varenicline use,^14^ and can produce estimates of cost-effectiveness very different to those estimated in a real-world context.^15^ Previous observational studies often lack matched controls and have been limited by restrictive exclusion criteria—such as excluding patients who received no or multiple pharmacotherapy^16^ or focusing on cohorts defined by a single diagnosis such as non-small cell lung cancer^17^—or by overly broad inclusion criteria, for example, combining inpatients and outpatients in analyses of hospital-initiated support.^18^

There is a need for real-world evidence of the cost and potential return on investment of hospital-initiated tobacco dependence treatment. One such study was an economic evaluation of a 6-month pilot of the Conversation, Understand, Replace, Experts and Evidence-based interventions (CURE) Project—an opt-out model similar to the OMSC—in Manchester, England. The estimated cost per quit was £475, based on the 22% quit rate of smokers followed up for 12 weeks after discharge,^10 19^ with an estimated gross financial return on investment of £2.12 per £1 invested over a payback period of 4 years. When using metrics which incorporate the value of improved patient health among those who quit smoking, the public value return on investment was £30.49 per £1 invested, and the cost per quality-adjusted life year (QALY) was £487.^19^

### Current study

In order to support the continued funding of OMSC-type interventions, further evidence is required to demonstrate effectiveness, both in terms of patient outcomes and cost. This exploratory study’s objectives were: (1) to estimate the provider and intervention delivery costs of an adapted OMSC intervention, implemented in an acute hospital in Southeast London, England, including the cost per patient, cost per quit and incremental cost effectiveness ratio (ICER) and (2) to estimate the variance in readmission costs and bed-days associated with the intervention by using patient-level readmission data from a matched cohort of patients who smoked but did *not* receive the intervention.

## Methods

### Setting

The study used data from 673 patients who smoked, admitted to one of 11 acute medical and surgical wards in a large teaching hospital run by King’s College Hospital NHS Foundation Trust in Southeast London, between 1 July 2020 and 30 June 2021 (herein referred to as the ‘OMSC group’). Median length of stay was 6 days. The 673 patients comprise all of the patients seen and assessed by the Tobacco Dependence Advisors (TDA) during their hospital stay. TDAs are specialists whose role is to deliver effective evidence-based tobacco dependence treatment in NHS inpatient settings, having undertaken practitioner training by the National Centre for Smoking Cessation and Training.^20^

Inpatient bed reconfiguration in response to the COVID-19 pandemic meant certain wards were not accessible during the study period and thus the OMSC group represents 40.2% of all identified smokers admitted to the hospital during the study period and 6.2% of all admissions. All patient data were recorded as part of routine clinical practice by the TDAs and other clinicians involved in patient care. Postdischarge phone calls were made by the TDAs to all 673 patients at 30-day intervals up to 6 months, except where a patient had specifically requested to opt out. Further details of the intervention and patient-related outcomes from a larger study involving this and another hospital Trust are described in Robins *et al*.^21^

### Economic evaluation

#### Cost per quit

The total provider and service delivery costs of running the adapted OMSC intervention were calculated and applied to the number of patients reporting being a non-smoker at 180 days after discharge. The ICER—the incremental cost per life year gained associated with the intervention—was calculated using the formula and tables provided in Stapleton and West.^22^ As postdischarge smoking status relied on self-report rather than biochemical verification, we calculated the cost per quit and ICER under a hypothetical ‘worst-case scenario’ as a sensitivity analysis. This incorporated a 40% lower quit rate and assumed an elevated background quit rate among hospitalised patients.

#### Patient-level provider and service delivery costs

The provider and service delivery costs of the intervention were estimated at a patient level using the NHS Trust’s Patient-Level Information and Costing Systems (PLICS) data, which combines activity, financial and operational data to cost individual episodes of patient care.^23^ The PLICS model calculates individual patients’ activity and resource usage and allocates overhead costs to derive the total cost of each patient episode recorded by the Trust’s Information department. The direct costs calculated were the full financial costs of the staffing establishment assigned to the project, with the costs of NRT and pharmacotherapy being itemised and matched to the patients in the cohort. Pharmacy staff costs were allocated using the Trust average cost per issue in the relevant financial period. Overhead costs comprised a proportion of corporate expenditure (eg, Finance, Human resources, Information and communications technology, Executives), allocated based on the number of staff and direct spend related to the project team. The process by which direct and overhead costs are calculated and quality assured in PLICS is described in detail elsewhere.^24^ As this was an intervention delivered by the NHS, there were no direct costs incurred by the patient when receiving the intervention.

#### Benchmarking

**Figure 1:**
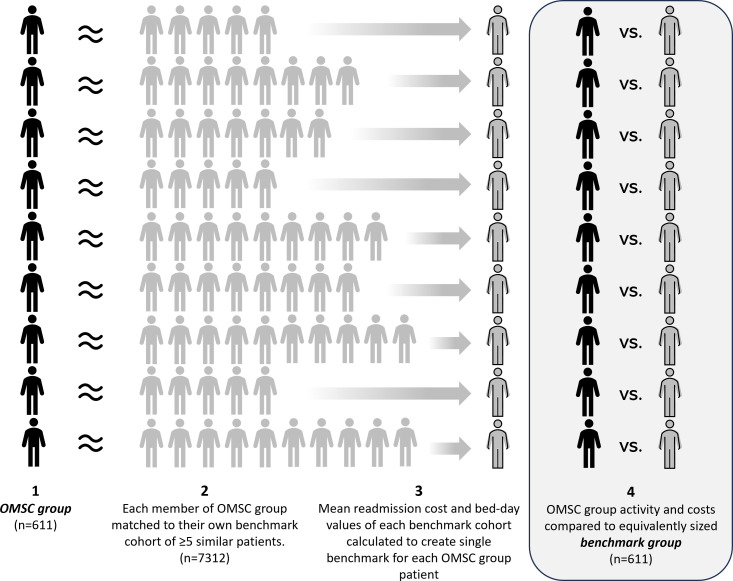
Benchmarking process: (1) after excluding patients with incomplete follow-up data or who could not be matched on PLICS, each patient in the OMSC group was matched to their own benchmark cohort, (2) consisting of at least 5 patients who did not receive the OMSC intervention but who were sufficiently similar in terms of demographic and clinical characteristics (ie, smoking status, how many smoked per day, Index of Multiple Deprivation decile, age, delivery (elective/emergency), Healthcare Resource Group root code and subchapter). (3) From each matched benchmark cohort, the mean readmission cost and bed-day values were calculated and used to represent the benchmark to which the readmission costs and bed-days of the OMSC patients were compared. (4) Collectively, these mean values form the ‘benchmark group’. OMSC, Ottawa Model for Smoking Cessation; PLICS, Patient-Level Information and Costing Systems.

The benchmarking focused on the frequency, cost and duration of patient readmissions, by comparing patients in the OMSC group to similar patients at the same hospital that had not received the adapted OMSC intervention, that is, patients who smoked but who had *not* been seen by a TDA. Comparing patient ID and the admission and discharge dates from the OMSC group to PLICS returned 653 costed patient spells, with 20 patients unmatched and discounted from the remaining calculation process. Patients who died within the year following discharge (n=22), or who had incomplete follow-up data (n=20), were also excluded from the analysis, leaving an analytic sample of 611 OMSC group patients. The benchmark patients were drawn from inpatient admissions between 1 July 2020 and 30 June 2021, after extreme outliers in terms of length of stay had been removed. Through data collected by clinicians on admission, we were able to identify patients that had declared themselves smokers. This dataset was then matched against the OMSC group data to find an appropriate benchmark cohort for each OMSC patient. See figure 1 for summary of benchmarking process.

#### Matching rules and loops

The OMSC patients were compared with the benchmark patients using combinations of the following criteria to ensure appropriate comparisons:

- How many cigarettes smoked per day, routinely recorded for all patients and categorised as 0–5, 6–10, 11–20, 21–30, 31+.
- Point of delivery code, a high-level means of classification for a commissioned healthcare activity, event or item (eg, elective inpatient/emergency attendance/non-elective short stay, etc)^25^
- Healthcare Resource Group root and subchapter code, from over 2600 codes representing the clinical classification of patient events that have been judged to consume a similar level of resource, based on procedure and diagnosis codes.^26^
- National decile of Index of Multiple Deprivation score, a measure of relative socioeconomic deprivation^27^
- 10-year age range, selected on the basis of being broad enough to return a benchmark cohort but without being too broad as to make the application of age-related criteria meaningless.

Additionally, a rule was set that a minimum of five comparable patients must be found in each benchmark cohort, to reduce the likelihood of the results being skewed by any one benchmark patient. These criteria were applied in 14 matching loops against the OMSC group to find the most appropriate benchmark cohort for each OMSC patient. Online supplemental Table S1 shows the matching process noting where each criterion has been matched or dropped per loop, as well as the number and proportion of OMSC group patients matched to cohorts in each loop.

#### Readmissions calculation

PLICS data were assessed for all patients across both cohorts to identify any readmissions after 180 days from original discharge from the Trust. The 180-day cut-off was chosen to ensure readmissions would be adjacent or subsequent to the final follow-up phone call to patients in the OMSC group, in which they would have reported their smoking status 6 months after discharge. It also focuses on a period when a patient’s reduction in smoking would be more likely to have a tangible effect on their health and likelihood of readmission.^28^ The PLICS data period for assessing readmissions covered the period from 1 January 2021 to 30 September 2022.

Readmission rates in the OMSC group were compared with those of the benchmark group, both overall and stratified by the self-reported smoking status at 6 months in the OMSC patients (ie, non-smoker, smoker, unknown or opted out). Comparisons by 6-month smoking status were only possible on the basis of the OMSC group smoking statuses, as the postdischarge smoking statuses of the benchmark group were unknown. Total readmission bed days and total readmission costs were calculated for the OMSC and benchmark groups, with the latter being derived using the mean values of each OMSC patient’s matched benchmark cohort. Bed days and costs per-patient-readmitted were calculated for the OMSC group and compared with those of the benchmark group, effectively comparing what the equivalent bed days and costs per-patient-readmitted would be from a group the same size as the OMSC group but with readmission rates, bed days and costs of the benchmark group.

### Patient involvement

Patients or the public were not involved in the design, conduct, reporting or dissemination plans of this study; however, patient participation and involvement group members contributed to the original programme of work from which this study derived.

## Results

673 patients who smoked were admitted to hospital and assessed by a TDA between 1 July 2020 and 30 June 2021. 44% (n=299) were successfully contacted at 6 months after discharge from hospital; 104 (15.5%) reported being a non-smoker and 195 (29%) reported still smoking. A further 37 patients (5.5%) had opted out of the intervention prior to the 6-month mark, and 295 (43.8%) were lost to follow-up (ie, did not answer the phone or provide a working phone number). The sociodemographic and clinical characteristics of the cohort are shown in online supplemental Table S2.

### Cost per quit

The total cost of the adapted OMSC programme between 1 July 2020 and 30 June 2021 was £178 105. This encompassed £130 061 staffing costs for three full-time equivalent TDAs and 0.1 full-time equivalent respiratory medical consultant, £15 893 NRT and pharmacotherapy costs (within 200 days of patient discharge), £21 637 pharmacy staff costs and £10 512 Trust overhead costs. Dividing by the 673 patients in the OMSC group results in a cost per patient of £264.64 and dividing by the 104 patients who reported being a non-smoker at 6 months after discharge results in a cost per quit of £1712.55. Using the tables and formula provided in Stapleton and West,^22^ the age-adjusted incremental cost per life year gained (ICER) was £3325 (see online supplemental material S3 for details of calculation).

#### Sensitivity analysis

We estimated the cost per quit and ICER under a hypothetical ‘worst-case scenario’ involving 40% lower quit rates and a 3.6% background quit rate. Under these circumstances, the cost per quit at 6 months would be £2873 per quit at 6 months, and age-adjusted ICER would be £9035 per life year gained (see online supplemental material S4 for details of calculation).

### Benchmark analysis

After excluding patients who died within the year following discharge (n=22), patients with missing follow-up data (n=20) and patients who could not be benchmarked (n=20), 611 patients from the OMSC group were included in the final benchmark analysis (mean age=50.6 years, SD=16.1). See figure 1 for details of benchmarking process. Of the OMSC group, 5.2% (n=32) were readmitted after 6 months from their discharge date. The benchmark cohorts consisted of 7312 matched patients in total (mean age=49.4, SD=18.2), of whom 11.1% were readmitted in the same timeframe (n=812).

The total readmission costs of the OMSC group were £266 288, and the averaged readmission costs from the benchmark group (n=611) were £757 811. This represents a total saving of £491 523 associated with the OMSC intervention. The OMSC group also incurred 414 fewer readmission bed days than the benchmark group (303 vs 717 bed days). In terms of per-patient-readmitted differences, the OMSC group had 1.2 fewer bed days and cost £2904.76 less per-patient-readmitted. See table 1 for comparison of total readmission rates, costs and bed days per patient readmitted for the OMSC group and benchmark group.

**Table 1:** Comparison of total readmission rates, costs and bed days per patient readmitted, for the OMSC group and the equivalently-sized benchmark group

Total activity numbers in OMSC and benchmark cohorts			
	OMSC	Benchmark	Variance
Patients	611	7312	
Total patients with readmissions	32	812	
**Readmission rate**	**5.2%**	**11.1%**	**−5.9%**

OMSC patients (n=611) versus benchmark group (n=611) patients			
	OMSC	Benchmark	Variance
Patients with readmissions	32	67.5	−35.5
Readmission bed days	303	717	−414
Bed days per patient readmitting	9.5	10.6	−1.1
Cost per patient readmitting (£)	£8321.50	£11 226.26	−£2904.76
Total readmissions cost (£)	£266 287.96	£757 811.20	−£491 523.24

OMSC, Ottawa Model for Smoking Cessation.

#### Stratification by 6-month quit status

**Figure 2:**
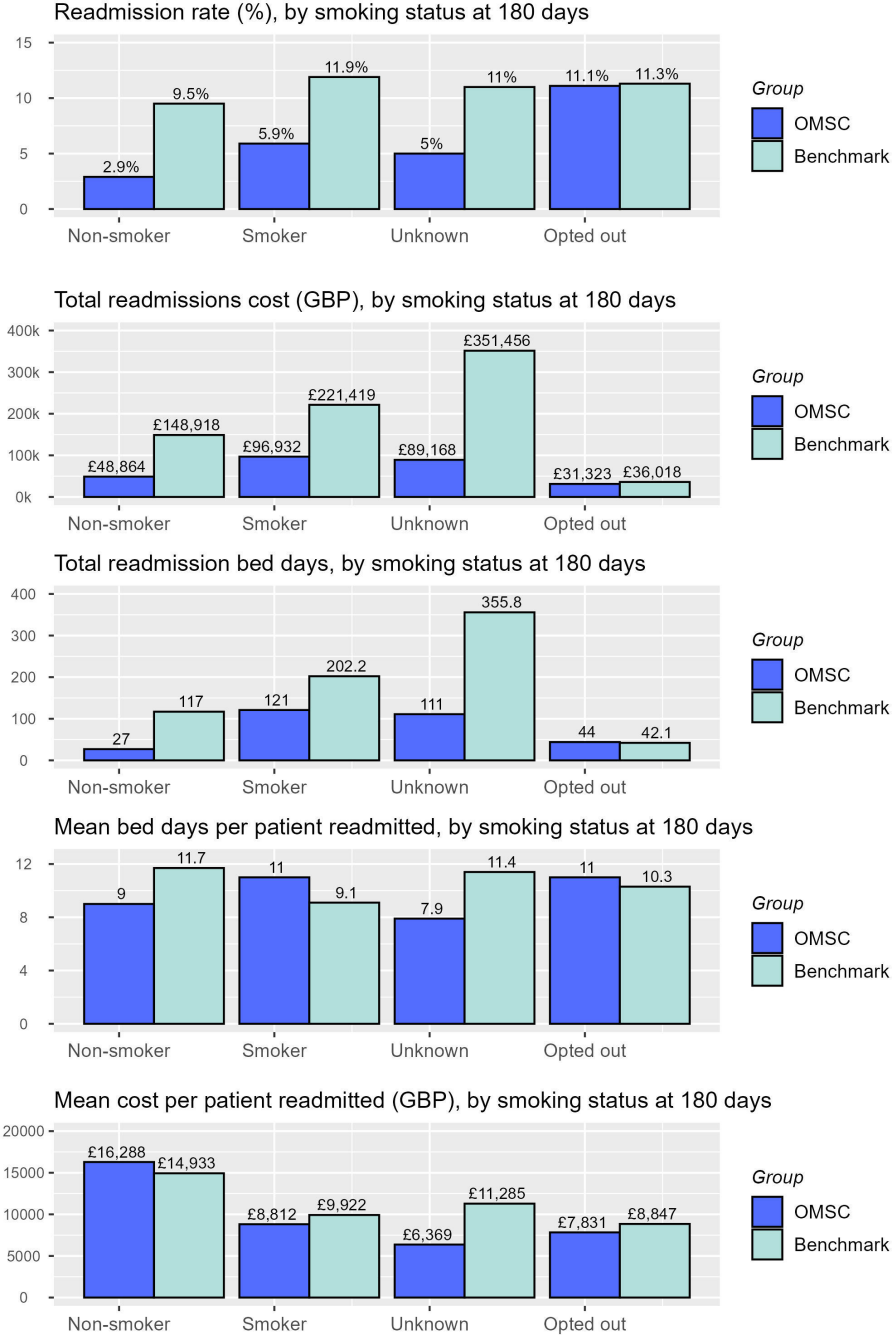
Comparisons between the OMSC group (n=611) and their benchmark group equivalents (n=611), according to OMSC group smoking status at 6 months after discharge: readmission rates (%), total readmissions cost (Great British Pound (GBP)), total readmission bed days, mean bed days per-patient-readmitted and mean cost per-patient-readmitted (GBP). OMSC, Ottawa Model for Smoking Cessation.

See figure 2 and table 2 for comparisons of the OMSC and benchmark groups in terms of readmission rates, total readmission costs and bed days and mean costs and bed days per-patient-readmitted, according to OMSC group smoking status at 6 months after discharge.

**Table 2:** Readmission rates, total readmissions costs and bed-days, mean costs and bed days per-patient-readmitted, stratified according to OMSC group smoking status at 6-month follow-up

OMSC group 6-month smoking status	Group	Readmission rate	Total readmissions cost	Total readmission bed days	Mean cost per-patient-readmitted	Mean bed-days per-patient-readmitted
**Non-smoker**	OMSC	2.9%	£48 864.18	27.0	£16 288.06	9.0
n=104	Benchmark	9.5%	£148 917.70	117.0	£14 933.48	11.7
	*Variance*	*−6.6%*	*−£100 053.52*	*−90.0*	*£1354.58*	*−2.7*
**Smoker**	OMSC	5.9%	£96 931.90	121.0	£8811.99	11.0
n=188	Benchmark	11.9%	£221 419.20	202.2	£9921.63	9.1
	*Variance*	*−6.0%*	*−£124 487.30*	*−81.2*	*−£1109.64*	*1.9*
**Unknown**	OMSC	5.0%	£89 168.41	111.0	£6369.17	7.9
n=282	Benchmark	11.0%	£351 456.20	355.8	£11 285.20	11.4
	*Variance*	*−6.1%*	*−£262 287.79*	*−244.8*	*−£4916.03*	*−3.5*
**Opted-out**	OMSC	11.1%	£31 323.47	44.0	£7830.87	11.0
n=37	Benchmark	11.3%	£36 018.08	42.1	£8846.55	10.3
	*Variance*	*−0.2%*	*−£4694.61*	*1.9*	*−£1015.68*	*0.7*
**All**	OMSC	5.2%	£266 288.00	303.0	£8321.50	9.5
n=611	Benchmark	11.1%	£757 811.20	717.0	£11 226.26	10.6
	*Variance*	*−5.9%*	*–£491 523.20*	*−414.0*	*−£2904.76*	*−1.2*

OMSC, Ottawa Model for Smoking Cessation.

When stratified according to OMSC group smoking status at 6 months, lower readmission rates and correspondingly lower costs were found among all smoking status categories for the OMSC group when compared with their benchmark group equivalents, except among those who opted out of the OMSC intervention, for whom there was no difference. Compared with their matched benchmarks, mean bed days per patient readmitted were lower for the non-smoker and unknown smoking status OMSC subgroups and marginally higher for the smoker and opt-out groups. Despite the cost per patient readmitted being highest for the non-smokers in the OMSC group and their benchmark equivalents, due to the difference in readmission rates between OMSC and benchmark groups, the OMSC group data still reveal significant cost savings. Of note, the mean age of the readmitted OMSC patients was highest in the non-smoking group (64.3 years (benchmark=61.7)) compared with readmitted patients in the smoking (53.9 years (benchmark=55.9)), unknown (54.0 years (benchmark=51.9)) and opt-out (57.0 years (benchmark=53.0)) groups.

## Discussion

### Summary of findings

An inpatient smoking cessation intervention based on the OMSC was implemented for 1 year in an acute hospital setting in London. Among 673 patients who were seen by a TDA, of the 299 who were able to be contacted 6 months after discharge, 104 reported being a non-smoker. The cost per quit was estimated to be £1713, with an age-adjusted ICER of £3325 per life year gained. Among the 611 patients who were able to be matched to a benchmark cohort of similar patients who did not receive the OMSC intervention, readmissions for patients in the OMSC group cost £492 k less than their benchmark equivalents for the 21 months from 1 January 2021 to 30 September 2022 (£266 k vs £758 k), incurred 414 fewer bed days (303 vs 717) and readmitted at a lower rate (5% vs 11%).

Applying the assumption used by the NHS—that approximately 50% of the fiscal benefits can be cashable if the scale of the transformation programme is large^19^—gives a fiscal benefit of £245 762 for the 21 months from 1 January 2021 to 30 September 2022, against the cost incurred for the intervention of £178 105 during the 12-month period from 1 July 2020 to 30 June 2021. This equates to a return on investment of £1.37 for every £1 spent. Considering this was a pilot intervention implemented during the unprecedented disruption of the 1 year of the COVID-19 pandemic in which over half of the admitted smokers were not seen by a TDA, suggests a potential for greater returns on investment following expansion of the intervention, with public health interventions typically generating greater cashable cost savings over longer periods of time and when implemented at scale.^29^ However, the costs of the OMSC intervention established here already place it well below the threshold of cost-effectiveness used by the National Institute of Health and Care Excellence in England; ICERs of less than £20 000 per QALY gained are considered cost-effective.^30^ Indeed, even under a hypothetical ‘worst-case scenario’ accounting for the absence of biochemical quit verification, and a potentially elevated background quit rate, the OMSC intervention costs remain well under this threshold. Furthermore, the financial benefits of quitting smoking accrue not only to the NHS but to the individual patients who quit, their families and wider society through increases in healthy life expectancy^2^ and reductions in health inequalities.^31^ In the London borough in which King’s College Hospital is situated, an estimated £85.6M is spent on tobacco, costing the borough £206M per year, with £11.2M spent on healthcare costs alone.^32^

Direct comparisons to other studies are difficult, due to variation in settings, type of patient, length of follow-up and modelling techniques.^12^ The closest comparison in terms of setting comes from the CURE Project pilot in Manchester, UK, which reported a lower cost per quit of £475 and ICER of £487 per QALY gained; however, this was based on quit rates at 12 weeks rather than 6 months after discharge and a cohort of more than double the size (n=1450).^19^ By contrast, a Dutch trial of an inpatient smoking cessation intervention for patients with CHD, followed up for 6 months, found approximately £4100 (€4781) of healthcare costs per quit, based on a group of 157 patients receiving face-to-face counselling.^33^

In our benchmarking analysis, the beneficial variance in readmission costs and bed days was primarily driven by reduced readmission rates in the OMSC group. While the greatest variance was found among those who reported not smoking a 6-month follow-up (2.9% vs 9.5% benchmark), similar reductions in readmission rate were found among those OMSC patients whose 6-month smoking status was unknown (5% vs 11% benchmark) and among those who reported still smoking (5.9% vs 11.9%). The only group for whom no meaningful reduction in readmission rate occurred were those patients who opted out of the OMSC intervention (11.1% vs 11.3% benchmark). While benefits derived in the unknown smoking status group might be due to some patients quitting smoking but not answering the phone to confirm, the benefits derived among the reported still-smoking group are more challenging to interpret and run counter to oft-used principle that no health benefit attributable to the intervention is assumed for those still smoking at final follow-up.^22^ However, it is possible that simply being exposed to the intervention prompts some future behaviour change resulting in reduced hospital readmission; for example, a trial of motivational tobacco cessation treatment combined with NRT in psychiatric inpatients found that being in the treatment group was associated with significantly decreased odds of rehospitalisation within 18 months compared with the usual-care control group, but actual quit success was not.^34^ It is also possible that the use of a point-prevalence measure of smoking status at 6 months conceals some quit success in the months leading up to that point, wherein a temporary relapse may have occurred.

A further unanticipated finding was that the mean costs per patient readmitted were highest among the OMSC patients who reported not smoking at 6-month follow-up and their benchmarks, compared with all other smoking status groups. However, this might be explained by the readmitted non-smokers being a particularly small group (n<10) with an older mean age (64 years) compared with readmissions in other smoking status categories (mean=55 years).

### Strengths and limitations

Our study uses a well-validated PLICS tool to attribute accurate healthcare costs to patient activity; an assessment of the Trust’s costing tool’s data quality and adherence to NHS Costing Transformation Programme standards was scored at 98% and has been ranked in the top five nationally.^35^ In terms of limitations, the estimated costs do not include postdischarge costs such as local authority Stop Smoking Services, to which some patients were referred, nor do they include wider societal costs such as lost productivity from being in hospital or higher pension costs due to increased lifespan.^36 37^ We do not know the reasons why the patients who opted out of the intervention chose to do so, as this was not systematically recorded. Postdischarge smoking status was not biochemically verified; however, this reflects the practical realities of treatment initiated during hospital admission and has been accounted for in our sensitivity analysis (see online supplemental material S4).^38^ We do not know the postdischarge smoking status of any of the benchmark patients, as they were not part of the TDA’s caseload, and so stratification according to 6-month smoking status is based purely on OMSC group smoking status. Delayed transfer of care could confound the readmission bed day calculations, although this risk applies equally across OMSC and benchmark groups, and so should not be a significant source of bias. Our study was conducted in an urban setting during the COVID-19 pandemic, and so, results may differ in different contexts.

## Conclusions

Our economic evaluation of an adapted Ottawa Model of Smoking Cessation intervention implemented in a major acute London hospital found the intervention to be sufficiently cost-effective. Reduced readmission rates and consequent costs were found for patients who received the intervention compared with their benchmarked equivalents, except for those who opted out.

## Supplementary material

10.1136/bmjopen-2025-107111 online supplemental file 1

## Data Availability

No data are available.
