# Supplementary material for: Economic evaluation of a hospital-initiated tobacco dependence treatment service
Source: BMJ Open. 2025 Dec 5;15(12):e107111. doi: 10.1136/bmjopen-2025-107111 (PMC12684108; doi:10.1136/bmjopen-2025-107111)
Supplement: online supplemental file 1 [file bmjopen-15-12-s001.doc]

Supplementary Material

S1: Matching rules and loops

|  | **Matching Loop Order Number** | | | | | | | | | | | | | |
| --- | --- | --- | --- | --- | --- | --- | --- | --- | --- | --- | --- | --- | --- | --- |
| **Matching rule** | **1** | **2** | **3** | **4** | **5** | **6** | **7** | **8** | **9** | **10** | **11** | **12** | **13** | **14** |
| **Smoker** | ✓ | ✓ | ✓ | ✓ | ✓ | ✓ | ✓ |  |  |  |  |  |  |  |
| **How Many Smoked** | ✓ | ✓ | ✓ | ✓ | ✓ | ✓ | ✓ | ✓ | ✓ | ✓ | ✓ | ✓ | ✓ | ✓ |
| **Point of Delivery** | ✓ | ✓ | ✓ | ✓ | ✓ | ✓ | ✓ | ✓ | ✓ | ✓ | ✓ | ✓ | ✓ | ✓ |
| **HRG Root** | ✓ | ✓ | ✓ | ✓ |  |  |  | ✓ | ✓ | ✓ | ✓ |  |  |  |
| **HRG Sub-Chapter** |  |  |  |  | ✓ | ✓ | ✓ |  |  |  |  | ✓ | ✓ | ✓ |
| **IMD Decile** | ✓ | ✓ |  |  | ✓ | ✓ |  | ✓ | ✓ |  |  | ✓ | ✓ |  |
| **Age Range** | ✓ |  | ✓ |  | ✓ |  | ✓ | ✓ |  | ✓ |  | ✓ |  | ✓ |
| **Min. 5 Spells in Bench cohort** | ✓ | ✓ | ✓ | ✓ | ✓ | ✓ | ✓ | ✓ | ✓ | ✓ | ✓ | ✓ | ✓ | ✓ |
| **% Patients Matched** | **7%** | **26%** | **7%** | **28%** | **1%** | **8%** | **3%** | **0%** | **1%** | **0%** | **10%** | **0%** | **2%** | **7%** |
| **Count of Patients Matched** | **45** | **158** | **44** | **169** | **6** | **47** | **19** | **0** | **8** | **0** | **59** | **0** | **15** | **41** |

***Supplementary Table S1:***

*The matching rules applied to patient records, used to identify benchmark cohorts matched to the OMSC group patients*

*HRG = Healthcare Resource Group (clinically meaningful groupings of patient activity derived from NHS patient records, primarily using procedure and diagnosis codes, see* [*www.digital.nhs.uk/services/secondary-uses-service-sus/payment-by-results-guidance/sus-pbr-reference-manual/hrg-grouping*](http://www.digital.nhs.uk/services/secondary-uses-service-sus/payment-by-results-guidance/sus-pbr-reference-manual/hrg-grouping)*); IMD = Index of Multiple Deprivation*

S2: Characteristics of OMSC group cohort

| **Variable** | **Category** | **n (%)** |
| --- | --- | --- |
| **Age** | 60+ | 228 (33.9%) |
|  | 40-59 | 260 (38.6%) |
|  | 25-39 | 144 (21.4%) |
|  | 16-24 | 41 (6.1%) |
| **Sex** | Male | 422 (62.7%) |
|  | Female | 251 (37.3%) |
| **Ethnicity** | Asian | 13 (1.9%) |
|  | Black | 125 (18.6%) |
|  | Mixed | 21 (3.1%) |
|  | Other | 63 (9.4%) |
|  | White | 389 (57.8%) |
|  | Declined or Not stated | 62 (9.2%) |
| **HSI category** | Low | 194 (28.8%) |
|  | Medium | 294 (43.7%) |
|  | High | 42 (6.2%) |
|  | *Missing* | *143 (21.2%)* |
| **Primary diagnosis** | Circulatory | 54 (8.0%) |
|  | Digestive | 99 (14.7%) |
|  | Endocrine and blood | 39 (5.8%) |
|  | Genitourinary | 31 (4.6%) |
|  | Infectious and parasitic | 26 (3.9%) |
|  | Injury poisoning and external | 179 (26.6%) |
|  | Mental Behavioural & Neurodevelopmental | 24 (3.6%) |
|  | Musculoskeletal | 20 (3.0%) |
|  | Neoplasms | 39 (5.8%) |
|  | Nervous system | 15 (2.2%) |
|  | Respiratory | 60 (8.9%) |
|  | Skin and subcutaneous | 12 (1.8%) |
|  | Other | 29 (4.3%) |
|  | *Missing* | *46 (6.8%)* |
| **IMD tertile** | Lower | 329 (48.9%) |
|  | Middle | 193 (28.7%) |
|  | Upper | 59 (8.8%) |
|  | *Missing* | *92 (13.7%)* |
| **Six-month smoking status** | Non-Smoker | 104 (15.5%) |
|  | Smoker | 195 (29.0%) |
|  | Unknown | 295 (43.8%) |
|  | Opted out | 37 (5.5%) |
|  | Patient deceased | 22 (3.3%) |
|  | *Missing* | *20 (3.0%)* |

**Supplementary Table S2**: Characteristics of cohort (N=673).

*IMD = Index of Multiple Deprivation, HSI = Heaviness of Smoking Index, Primary diagnoses grouped according to ICD-10 chapter*

### S3: Incremental Cost-Effectiveness Ratio

Incremental Cost-Effectiveness Ratio = the ratio of the increased cost per person to provide the intervention (incremental cost, IC) and the health benefit per person (incremental effectiveness, IE): ICER = IC/IE.

**Intervention effect** =

- 104 non-smokers at 6 months / 673 in cohort = 15.5%
- Minus 2.5% background quit rate in general population = 13%

**Intervention cost** = £264.64 per smoker treated

**ICER interpolated from Table 3A in (Stapleton & West, 2012)** = £2634

**Adjustment for age** **distribution in OMSC group** (see Stapleton & West, 2012) =

- Over 54 = 47.1% (multiply ICER by 1.36)
- 35-54 = 32.7% (ICER as above)
- Under 35 = 20.2% (multiply ICER by 1.46)

**Put it all together =**

- 0.202 * (1.46 * 2634) + 0.327 * 2634 + 0.471 * (1.36 * 2634) = **£3325.37**

***So this would mean the OMSC intervention costs £1712.55 per quit at 6 months, and £3325 per Life Year gained.***

**Assumptions:**

- assumes that there is no benefit to anything other than permanent cessation, which is unlikely to be true
- assumes that all of the 295 patients whose smoking status was unknown at six months follow-up continued to smoke
- assumes 3.5% discounting rate (i.e. later years = less value)
- assumes that 2.5% of smokers would have quit anyway, regardless of intervention
- assumes 48.75% of those abstinent at 6 months will remain so (others relapse)

**See:**

Stapleton, J. A., & West, R. (2012). A Direct Method and ICER Tables for the Estimation of the Cost-Effectiveness of Smoking Cessation Interventions in General Populations: Application to a New Cytisine Trial and Other Examples. *Nicotine & Tobacco Research*, 14(4), 463–471. <https://doi.org/10.1093/ntr/ntr236>

### S4: ICER estimated under worst-case scenario

The above calculation (S3) can be repeated under conditions of a highly conservative ‘worst-case scenario’, in which it is assumed that:

- the intervention quit rate is lower, to account for potential discrepancies between self-reported and CO-validated quits
- the background quit rate is higher, to reflect the potential for a higher proportion of patients quitting after discharge from hospital than in the general population of smokers not accessing hospital or community tobacco dependence treatment

**Adjusting for lower CO-validated quit rate**

Evison et al.’s evaluation of the CURE pilot intervention in Manchester, England, included opportunity for patients to attend a face-to-face consultation at four weeks after discharge; of the 398 who attended, 293 validated their successful quit via exhaled CO measurement. This represents 12% of all admitted patients who smoked (n=293/2393), a 40% reduction compared to the 21% who self-reported quitting (n=495/2393) [1].

Applying the same percentage reduction to our quit rate reduces it from 15.5% to 9.3%.

**Adjusting for a higher background quit rate**

There are few data on the background quit rate of patients discharged from an acute hospital admission. In a cluster RCT by Murray et al. in Nottingham, England, the usual care condition consisted of opportunistic brief advice from ward staff, with no systematic identification of smokers, no pharmacotherapy, and no post-discharge follow-up. At six months, 8.3% (n=19/229) were abstinent in the usual care condition, compared to 17.8% (n=47/264) in the structured intervention arm that included specialist support, medication, and follow-up [2].

Applying the same relative percentage difference between intervention and background quit rates increases the background quit rate from 2.5% to 3.6%.

**Applying these values to our calculation:**

**Intervention effect** =

- 62 non-smokers at 6 months / 673 in cohort = 9.3%
- Minus 3.6% background quit rate in patients discharged from hospital = 5.7%

**Intervention cost** = £264.64 per smoker treated

**ICER interpolated from Table 3A in Stapleton & West, 2012** [3] = £7157

**Applying age adjustment =**

- 0.202 * (1.46 * 7157) + 0.327 * 7157 + 0.471 * (1.36 * 7157) = **£9035.60**

***So this would mean under an estimated ‘worst-case scenario’, the OMSC intervention would cost £2873 per quit at 6 months, and £9035 per Life Year gained.***

**Caveats**:

- All previously mentioned conservative assumptions still apply (e.g. that all patients whose smoking status was unknown at six months continued to smoke, and that there is no benefit to anything other than permanent cessation, both of which are unlikely to be true)
- Discrepancies between self-reported and CO validated quit rates vary wildly, from 1-47%, and in some cases go the other way, with CO validation finding higher quit rates than self-report [4]
- The background quit rate amongst patients discharged from an acute hospital admission without receiving tobacco dependence treatment is highly speculative as there are no good quality data; indeed, there are some data which suggest that the background quit rate amongst this population may be lower than that of the general population of smokers not accessing hospital or community tobacco dependence treatment [5].

[1] Evison M, Pearse C, Howle F, Baugh M, Huddart H, Ashton E, et al. Feasibility, uptake and impact of a hospital-wide tobacco addiction treatment pathway: Results from the CURE project pilot. Clin Med (Northfield Il) [Internet]. 2020 Mar 18;20(2):196–202. Available from: https://www.rcpjournals.org/lookup/doi/10.7861/clinmed.2019-0336

[2] Murray RL, Leonardi-Bee J, Marsh J, Jayes L, Li J, Parrott S, et al. Systematic identification and treatment of smokers by hospital based cessation practitioners in a secondary care setting: cluster randomised controlled trial. BMJ [Internet]. 2013 Jul 8;347(jul08 1):f4004–f4004. Available from: https://www.bmj.com/lookup/doi/10.1136/bmj.f4004

[3] Stapleton JA, West R. A Direct Method and ICER Tables for the Estimation of the Cost-Effectiveness of Smoking Cessation Interventions in General Populations: Application to a New Cytisine Trial and Other Examples. Nicotine Tob Res [Internet]. 2012 Apr 1;14(4):463–71. Available from: https://academic.oup.com/ntr/article-lookup/doi/10.1093/ntr/ntr236

[4] Gorber SC, Schofield-Hurwitz S, Hardt J, Levasseur G, Tremblay M. The accuracy of self-reported smoking: A systematic review of the relationship between self-reported and cotinine-assessed smoking status. Nicotine Tob Res [Internet]. 2009 Jan;11(1):12–24. Available from: https://academic.oup.com/ntr/article-lookup/doi/10.1093/ntr/ntn010

[5] Devani N, Mangera Z, Smith H, Gates J, Woodhouse A, Fullerton D, et al. ‘The dark before the dawn’: the 2021 British Thoracic Society Audit of the treatment of tobacco dependency in acute trusts. BMJ Open Respir Res [Internet]. 2023 Nov 29;10(1):e001532. Available from: https://bmjopenrespres.bmj.com/lookup/doi/10.1136/bmjresp-2022-001532
