# Supplementary material for: Economic evaluation of a hospital-initiated tobacco dependence treatment service
Source: BMJ Open. 2025 Dec 5;15(12):e107111. doi: 10.1136/bmjopen-2025-107111 (PMC12684108; doi:10.1136/bmjopen-2025-107111)
Supplement: Supplementary file 2 [file bmjopen-2025-107111.reviewer_comments.pdf]

PEER REVIEW HISTORY

BMJ Open publishes all reviews undertaken for accepted manuscripts. Reviewers are asked to complete a checklist review form and are provided with free text boxes to elaborate on their assessment. These free text comments are reproduced below.

ARTICLE DETAILS

Title (Provisional)

Economic evaluation of a hospital-initiated tobacco dependence treatment service

Authors

Robins, John; Alltimes, Gary; Patel, Irem; McNeill, Ann; Moxham, J; Duckworth Porras, Stephanie; Stock, Andrew; Woodhouse, Arran; Robson, Deborah

---

VERSION 1 - REVIEW

**Reviewer**	**1**
**Name**	**Gawde, Nilesh**
**Affiliation**	**Tata Institute of Social Sciences**
**Date**	**02-Aug-2025**
**COI**	**None**

The paper Economic evaluation of a hospital-initiated tobacco dependence treatment service is of merit and adds value to the existing knowledge on effectiveness and cost-effectiveness of smoking cessation interventions in hospital settings.

The methods are in line with scientific principles and the framework for economic evaluations. The description and follow-up of the intervention group, calculation of effects and readmission costs is appropriate in my view. The selection of benchmark group for cost comparison for readmissions follows rational method.

The manuscript needs minor revisions which are listed below.

The authors have used STROBE checklist which is useful for observational studies. This study is an interventional design and has economic evaluation based on data from the intervention. Hence, CHEERS statement will be more appropriate. Please do the needful.

Total cost of the intervention is mentioned as £178,105. The break-up indicates that it is provider cost. Did the costs include costs incurred by patients when they used the intervention (direct or indirect costs)? If the patient costs were not included, whether it was because these were negligible or because the economic evaluation was with a provider perspective and not societal perspective?

Was the costing financial (budgets spent for the intervention by provider) or economical one (costs not accounted for in budget such as infrastructure of the hospital) were also included? The authors have mentioned about overhead costs but this was not clear to me.

The difference in costs of readmissions was predominantly due to the difference in the readmission rates. There was a large benchmarking group and the matching process seems robust. However, the period Jan 21 to Sep 23 was disrupted by waves of corona pandemic and the admissions were affected directly due to covid infections and indirectly due to exacerbation of chronic conditions. Since the data includes only 32 cases of readmission, there is a concern whether this group and the benchmarking group had variations due to the pandemic. It was clear that the intervention group is from a single hospital in a locality but the benchmarking group seems to be from within NHS (not clear whether in London or elsewhere in UK).

Supplementary file (S3) mentions 2.5% background quit rate in general population. It is known that hospitalisation episodes can lead to quitting as the patients receive information about their condition and advice from healthcare providers and also from other sources. What is the background quit rates post-hospitalisation in UK. That may be useful for sensitivity analysis for data presented in the supplementary file S3.

Ethical approval: While the authors have mentioned that they have used data routinely captured in electronic health records, the data includes that from an intervention where persons were followed up to six-month post-discharge and whether that part of data collection was covered with IRB approval is unclear. Editor to please take a call on this point.

**Reviewer**	**2**
**Name**	**Goel, Sonu**
**Affiliation**	**PGIMER, SCHOOL OF PUBLIC HEALTH**
**Date**	**25-Aug-2025**
**COI**	**None**

This manuscript presents an economic evaluation of an adapted Ottawa Model for Smoking Cessation (OMSC) intervention delivered in an acute hospital in London. Using Patient-Level Information and Costing Systems (PLICS) data, the study estimates cost per quit, incremental cost-effectiveness ratio (ICER), and return on investment, comparing patients who received the intervention to a matched benchmark cohort of smokers who did not. The study addresses a key evidence gap on cost-effectiveness of hospital-initiated smoking cessation services in the UK.

The paper is well-written, relevant to NHS policy, and uses robust cost data. However, there are important methodological and interpretive issues that should be addressed before acceptance. The comments are attached in the word file.

Summary
This manuscript presents an economic evaluation of an adapted Ottawa Model for Smoking Cessation (OMSC) intervention delivered in an acute hospital in London. Using Patient-Level Information and Costing Systems (PLICS) data, the study estimates cost per quit, incremental cost-effectiveness ratio (ICER), and return on investment, comparing patients who received the intervention to a matched benchmark cohort of smokers who did not. The study addresses a key evidence gap on cost-effectiveness of hospital-initiated smoking cessation services in the UK.
The paper is well-written, relevant to NHS policy, and uses robust cost data. However, there are important methodological and interpretive issues that should be addressed before acceptance.

Reviewer Comments

Abstract
1. Include ICER value in result section for more clearity.

Key Messages
2. Clerify the time frame and assumptionsused in RIC calculation for better understanding.

Background

3. The gap in the literature is not clearly articulated. Please highlight the specific gap that this study addresses.
4. The objectives are not clearly stated in the background. They should be explicitly mentioned to align with the study's purpose.
5. Line 59, reference should be of same format.

Methods

6. Clarify how the sample size was determined. A brief explanation or reference would strengthen the methods section.
7. Describe the patient selection and sampling strategy in more detail, so the reader understands how participants were recruited.
8. Provide more details on data collection: such as Who collected the data? Whether data collectors were trained?
9. How was data quality assured (e.g., through checks or coding procedures)?
10. Clerify about the remaining 60% (refer to line 85)
11. Line 87, better to give the characteristics/reasons for the opt-out population.
12. The variables in *Matching rules & loops section* can be discussed briefly in the section
13. The dates and the timeline should be uniform in the whole manuscript.
14. Line 145, check the referred fgure and should be in result section.
15. Ethical clearance details should be included at the end of the Methods section (mention institutional approval and reference number if available).

Results
16. It needs to be clarified in the Results section why 295 patients were not contacted (non-response, loss to follow-up, refusal, etc.).
17. Consider combining Figures 2 and 3 into a single comparative bar chart for easier visualisation and clearer comparison.

18. Use some Model for the signicance results in Table 2 , (p-value and CI)

Discussion
19. In line 232, check the grammer mistake.

Strengths and Limitation
20. Regarding the societal cost, you can mention from previous literature.
21. As youmentioned in the limitation that smiking status was not biochemically varified, it means self-report may overestimate quit rates. In discussion section, you can mention how the cost per quit changes if fewer people actually quit than reporting. Additionally, You can add whether results might differ in Non-Urban or post pandemic settings.

VERSION 1 - AUTHOR RESPONSE

Reviewer	Number	Issue	Response/Action
**Reviewer 1**	1.1	The authors have used STROBE checklist which is useful for observational studies. This study is an interventional design and has economic evaluation based on data from the intervention. Hence, CHEERS statement will be more appropriate. Please do the needful.	We have now completed the CHEERS checklist as requested.
	1.2	Total cost of the intervention is mentioned as £178,105. The break-up indicates that it is provider cost. Did the costs include costs incurred by patients when they used the intervention (direct or indirect costs)? If the patient costs were not included, whether it was because these were negligible or because the economic evaluation was with a provider perspective and not societal perspective?	We thank the reviewer for bringing this need for further clarity to our attention. The total cost reported is indeed purely provider and service delivery costs. We have used tried and tested model that is accepted in the UK, which is wider than just provider costs as it also includes pharma and indirect costs for patient care. However, we have not done a full societal model which takes into account lost productivity from being ill, etc., and we do report this in the limitations.<br><br>Patient costs were not included as NHS interventions are free for the patient at the point of delivery.<br><br>We have made the following amendments to the manuscript:<br>• Methods, line 125: The first sentence under the 'Cost per quit' sub-heading now reads "*The total provider and service delivery costs of running the adapted OMSC intervention…*"

Reviewer	Number	Issue	Response/Action
			• Methods, line 133: sub-heading ‘Patient-level costs’ now reads ‘Patient-level provider and service delivery costs;<br>• Methods, line 134: The first sentence now reads “*The provider and service delivery costs of the intervention were estimated at a patient level using…*”<br>• Methods, line 145: We have added the sentence “*As this was an intervention delivered by the NHS, there were no direct costs incurred by the patient when receiving the intervention*”.
	1.3	Was the costing financial (budgets spent for the intervention by provider) or economical one (costs not accounted for in budget such as infrastructure of the hospital) were also included? The authors have mentioned about overhead costs but this was not clear to me.	We have added further detail about the type of costs included in the PLICS model, under the ‘Patient-level provider and service delivery costs’ subheading in the Methods:<br><br>“*The provider and service delivery costs of the intervention were estimated at a patient level using the NHS Trust’s Patient-Level Information and Costing Systems (PLICS) data, which combines activity, financial, and operational data to cost individual episodes of patient care (23). The PLICS model calculates individual patients’ activity and resource usage and allocates overhead costs to derive the total cost of each patient episode recorded by the Trust’s Information department. The direct costs calculated were the full financial costs of the staffing establishment assigned to the project, with the costs of NRT and pharmacotherapy being itemised and matched to the patients in the cohort. Pharmacy staff costs were allocated using the Trust average cost per issue in the relevant financial period. Overhead costs comprised a proportion of corporate expenditure (e.g. Finance, HR, ICT, Executives), allocated based on the number of staff and direct spend related to the project team. The process by which direct and overhead costs are calculated and quality assured in PLICS is described in detail elsewhere (24). As this was an intervention delivered by the NHS, there were no direct costs incurred by the patient when receiving the intervention*.” (Lines 133–146)
	1.4	The difference in costs of readmissions was predominantly due to the difference in the readmission rates. There was a large	We thank the reviewer for their thoughtful concern, and identifying the need for clarity here. All of the patients which formed the benchmarking group were in fact from the same hospital as the patients receiving the OMSC intervention, so although readmission rates may have been affected by the

Reviewer	Number	Issue	Response/Action
		benchmarking group and the matching process seems robust. However, the period Jan 21 to Sep 23 was disrupted by waves of corona pandemic and the admissions were affected directly due to covid infections and indirectly due to exacerbation of chronic conditions. Since the data includes only 32 cases of readmission, there is a concern whether this group and the benchmarking group had variations due to the pandemic. It was clear that the intervention group is from a single hospital in a locality but the benchmarking group seems to be from within NHS (not clear whether in London or elsewhere in UK).	COVID pandemic, this should have applied equally to both the intervention and benchmarking groups.<br><br>To make it clearer that both patient groups came from the same hospital we have amended the first sentence under the 'Benchmarking' subheading in the Methods, so that it now reads as follows (Lines 148-150):<br><br>*"The benchmarking focussed on the frequency, cost, and duration of patient readmissions, by comparing patients in the OMSC group to similar patients at the same hospital that had not received the adapted OMSC intervention, i.e. patients who smoked but who had not been seen by a TDA."*
	1.5	Supplementary file (S3) mentions 2.5% background quit rate in general population. It is known that hospitalisation episodes can lead to quitting as the patients receive information about their condition and advice from healthcare providers and also from other sources. What is the background quit rates post-hospitalisation in UK. That may be useful for sensitivity analysis for data presented in	This is a valid concern. There are few reliable data concerning the background quit rate amongst smokers discharged from hospital in the UK without receiving a tobacco dependence treatment intervention; indeed a 2021 British Thoracic Society national audit of 119 hospitals suggested that it may in fact be lower than the general population (under 1%), see Devani et al (2023)<br><br>However, we have endeavoured to estimate a 'worst-case scenario' cost per quit and ICER, incorporating a 3.6% (rather than 2.5%) background quit rate derived from Murray et al.'s 2023 RCT in Nottingham, England, and a 40% reduced intervention quit rate based on an assumption that CO-validated quit rates would be lower than self-reported quit rates (in response to a concern from reviewer 2). These calculations are outlined in detail in a new section of the supplementary material (see section S4), and have been included in the main manuscript Methods (Lines

Reviewer	Number	Issue	Response/Action
		the supplementary file S3.	129-132), Results (Lines 227-231), and Discussion (Lines 291-294).<br><br>Devani N, Mangera Z, Smith H, Gates J, Woodhouse A, Fullerton D, et al. 'The dark before the dawn': the 2021 British Thoracic Society Audit of the treatment of tobacco dependency in acute trusts. BMJ Open Respir Res. 2023 Nov 29;10(1):e001532.<br><br>Murray RL, Leonardi-Bee J, Marsh J, Jayes L, Li J, Parrott S, et al. Systematic identification and treatment of smokers by hospital based cessation practitioners in a secondary care setting: cluster randomised controlled trial. BMJ. 2013 Jul 8;347(jul08 1):f4004–f4004.
	1.6	Ethical approval: While the authors have mentioned that they have used data routinely captured in electronic health records, the data includes that from an intervention where persons were followed up to six-month post-discharge and whether that part of data collection was covered with IRB approval is unclear. Editor to please take a call on this point.	Thank you for pointing this out. We have now included an 'Ethical approval' section at the end of the Methods, which reads as follows:<br><br>*"As this was a service evaluation using anonymised data routinely collected as part of a clinical service, ethical approval was not required (18). Service evaluation approval was obtained from King's College Hospital NHS Foundation Trust prior to the evaluation (reference 11641: Service evaluation and clinical audit of tobacco dependence treatment started in hospital)."* (Lines 204-208)<br><br>The follow-up phone calls are a core part of the Ottawa Model for Smoking Cessation itself, and the recording of smoking status at each follow-up point comes under the umbrella of 'routinely collected data', and therefore did not require any separate approval beyond that described above.
**Reviewer 2**	2.1	Abstract<br>Include ICER value in result section for more clarity.	We have now included the ICER value in the results section. The second sentence of this section now reads "*On the basis of 104 patients who reported not smoking at six months, the cost-per-quit was £1712.55, equating to an estimated age-adjusted incremental cost per life year gained of £3325.*" (Line 26)
	2.2	Key Messages<br>Clarify the time frame and assumptions used in RIC calculation for better understanding.	As per BMJ Open guidelines the 'Key messages' section has been removed
	2.3	Background<br>The gap in the literature is not clearly articulated. Please highlight the specific	We have added further detail to the third paragraph of the Background section, which now reads as follows (Lines 74–87):

Reviewer	Number	Issue	Response/Action
		gap that this study addresses.	*"There is less evidence regarding the cost-effectiveness of hospital-initiated smoking cessation interventions, although where such research has been conducted the results are favourable, despite differences in delivery of the intervention and the methods of evaluation (12). The majority of the current evidence comes from the USA, which uses a different model of health care funding to countries such as the UK, the latter being taxpayer-funded and free at the point of use (13). Many of the health economic analyses of tobacco dependence treatment also come from randomised controlled trials, which are often concerned with the cost effectiveness of a single element of an intervention compared to its closest alternative, e.g. standard versus enhanced varenicline use (14), and can produce estimates of cost-effectiveness very different to those estimated in a real-world context (15). Previous observational studies often lack matched controls and have been limited by restrictive exclusion criteria—such as excluding patients who received no or multiple pharmacotherapy (16) or focusing on cohorts defined by a single diagnosis such as non-small cell lung cancer (17)—or by overly broad inclusion criteria, for example combining inpatients and outpatients in analyses of hospital-initiated support (18)."*
	2.4	Background<br>The objectives are not clearly stated in the background. They should be explicitly mentioned to align with the study's purpose.	We have amended the 'Current study' paragraph at the end of the Background section, such that it now reads as follows (Lines 97–104):<br><br>*"In order to support the continued funding of OMSC-type interventions, further evidence is required to demonstrate effectiveness, both in terms of patient outcomes and cost. This exploratory study's objectives were 1) to estimate the provider and intervention delivery costs of an adapted OMSC intervention, implemented in an acute hospital in Southeast London, England, including the cost per patient, cost per quit, and Incremental Cost Effectiveness Ratio (ICER), and 2) to estimate the variance in readmission costs and bed-days associated with the intervention by using patient-level readmission data from a matched cohort of patients who smoked but did not receive the intervention."*
	2.5	Background<br>Line 59, reference should be of same format.	We are not sure what discrepancy of format the reviewer is referring to here. The text in question reads "*...quit attempts and quit success (5–10) and reducing readmission rates and mortality (5,11)*" (Line 73)

Reviewer	Number	Issue	Response/Action
			This obeys the BMJ reference style guide, which states that multiple reference citations should be separated by commas (5,11) or by hyphens if numbers are sequential (5-10) (see https://authors.bmj.com/writing-and-formatting/formatting-your-paper/)
	2.6	Methods<br>Clarify how the sample size was determined. A brief explanation or reference would strengthen the methods section.	As this study was part of a pragmatic service evaluation of a pilot intervention that we previously published (Robins et al 2024) all patients who smoked were eligible for inclusion, and no formal sample size calculations were conducted. Due to inpatient bed reconfiguration in response to the COVID-19 pandemic, certain wards were not accessible by the Tobacco Dependence Advisors and so the included patients do not represent 100% of the smokers admitted to the hospital, however they do comprise 100% of the smokers who were seen and assessed by the Tobacco Dependence Advisors.<br><br>We thank for reviewer for bringing our attention to how the ambiguity in how this was previously reported, have now added further elaboration to the 'Setting' section of the Methods, to make it clearer that the included patients were not recruited on the basis of any patient characteristic (other than being a current smoker and being accessible by the Tobacco Dependence Advisors). The manuscript now reads as follows (Lines 110–111 and 115-117):<br><br>"*The 673 patients comprise all of the patients seen and assessed by the Tobacco Dependence Advisors (TDA) during their hospital stay…*<br>*…Inpatient bed reconfiguration in response to the COVID-19 pandemic meant certain wards were not accessible during the study period and thus the OMSC group represents 40.2% of all identified smokers admitted to the hospital during the study period, and 6.2% of all admissions."*<br><br>Robins J, Patel I, McNeill A, Moxham J, Woodhouse A, Absalom G, et al. Evaluation of a hospital-initiated tobacco dependence treatment service: uptake, smoking cessation, readmission and mortality. BMC Med [Internet]. 2024 Mar 25;22(1):139. Available from: https://doi.org/10.1186/s12916-024-03353-8
	2.7	Methods<br>Describe the patient selection and sampling	See response to 2.6 above

Reviewer	Number	Issue	Response/Action
		strategy in more detail, so the reader understands how participants were recruited.	
	2.8	Methods<br>Provide more details on data collection: such as Who collected the data? Whether data collectors were trained?	All patient data were collected by clinicians as part of routine clinical practice. Data specifically related to tobacco dependence treatment were recorded by the Tobacco Dependence Advisors (TDA) as part of their routine clinical practice. All TDAs are trained to a minimum standard by the National Centre for Smoking Cessation and Training (https://www.ncsct.co.uk/publications/category/inpatient-acute-training-resources)<br><br>We have added further detail to the 'Setting' paragraph of the Methods, as follows:<br><br>"*TDAs are specialists whose role is to deliver effective evidence-based tobacco dependence treatment in NHS inpatient settings, having undertaken practitioner training by the National Centre for Smoking Cessation and Training (NCSCT) (20).*" (Lines 111– 114)<br><br>"*All patient data were recorded as part of routine clinical practice by the TDAs and other clinicians involved in patient care. Post-discharge phone calls were made by the TDAs to all 673 patients at 30-day intervals up to six months, except where a patient had specifically requested to opt-out.*" (Lines 117–120)
	2.9	Methods<br>How was data quality assured (e.g., through checks or coding procedures)?	Data relating to the delivery of the OMSC intervention and follow-up phone calls were directly recorded on the hospital electronic health record systems by the TDAs after patient contact.<br><br>The activity data used in PLICS comes from the Trust's Business Informatics department and are subject to rigorous data quality and coding checks as part of the monthly data cycle. This includes any data challenges from commissioners. The data used in the PLICS model is processed after all of these data checks have taken place. The Costing team then applies sense checks based on the monthly count and casemix of all inputs, and cost centre allocation rules are reviewed

Reviewer	Number	Issue	Response/Action
			with finance teams regularly to ensure relevance and accuracy. The Costing team also carry out post-calculation data validation on the PLICS outputs before publishing.<br>In terms of benchmarking, we ensured relevance and accuracy by creating a bespoke benchmarking cohort for each patient which covered a combination of primary condition (HRG root/sub-chapter), age range, IMD decile and smoking status to ensure like-for-like comparisons. Outliers were removed to avoid skewing values in the benchmark cohort.<br><br>We have provided further description of the PLICS data in the manuscript and included a reference to further details by which the direct and overhead costs are calculated and quality assured (Lines 133-146):<br><br>*"The provider and service delivery costs of the intervention were estimated at a patient level using the NHS Trust's Patient-Level Information and Costing Systems (PLICS) data, which combines activity, financial, and operational data to cost individual episodes of patient care (23). The PLICS model calculates individual patients' activity and resource usage and allocates overhead costs to derive the total cost of each patient episode recorded by the Trust's Information department. The direct costs calculated were the full financial costs of the staffing establishment assigned to the project, with the costs of NRT and pharmacotherapy being itemised and matched to the patients in the cohort. Pharmacy staff costs were allocated using the Trust average cost per issue in the relevant financial period. Overhead costs comprised a proportion of corporate expenditure (e.g. Finance, HR, ICT, Executives), allocated based on the number of staff and direct spend related to the project team. The process by which direct and overhead costs are calculated and quality assured in PLICS is described in detail elsewhere (24). As this was an intervention delivered by the NHS, there were no direct costs incurred by the patient when receiving the intervention."*
	2.10	Methods<br>Clarify about the remaining 60% (refer to line 85)	As mentioned in response to point 2.6 above, further elaboration to the 'Setting' section of the Methods has now been included:

Reviewer	Number	Issue	Response/Action
			"The 673 patients comprise all of the patients seen and assessed by the Tobacco Dependence Advisors (TDA) during their hospital stay... *...Inpatient bed reconfiguration in response to the COVID-19 pandemic meant certain wards were not accessible during the study period and thus the OMSC group represents 40.2% of all identified smokers admitted to the hospital during the study period, and 6.2% of all admissions."* (Lines 110–111 and 115-117)
	2.11	Methods Line 87, better to give the characteristics/reasons for the opt-out population.	Unfortunately, reasons for opting out of the OMSC intervention were not routinely recorded at the time, and so we do have these data. <br>We thank the reviewer for highlighting this limitation and have now added a sentence to the 'Strengths and Limitations' section of the Discussion accordingly: <br>"*We do not know the reasons why the patients who opted-out of the intervention chose to do so, as this was not systematically recorded*." (Lines 338–339)
	2.12	Methods The variables in Matching rules & loops section can be discussed briefly in the Section	As requested we have now added more description of the variables in the 'Matching rules & loops' section of the Methods (Lines 161–174): <br>*"The OMSC patients were compared to the benchmark patients using combinations of the following criteria to ensure appropriate comparisons:* • *how many cigarettes smoked per day, routinely recorded for all patients, and categorised as 0-5, 6-10, 11-20, 21-30, 31+.* • *point of delivery code, a high-level means of classification for a commissioned health care activity, event or item (e.g. elective inpatient / emergency attendance / non elective short stay, etc.) (25)* • *Healthcare Resource Group (HRG) root and sub-chapter code, from over 2,600 codes representing the clinical classification of patient events that have been judged to consume a similar level of resource, based on procedure and diagnosis codes (26)* • *national decile of Index of Multiple Deprivation (IMD) score, a measure of relative socio-economic deprivation (27)* • *ten-year age range, selected on the basis of being broad enough to return a benchmark cohort but without being too broad as to make the application of age-related criteria meaningless."*

Reviewer	Number	Issue	Response/Action
	2.13	Methods<br>The dates and the timeline should be uniform in the whole manuscript.	We have checked and made dates consistent throughout the manuscript
	2.14	Methods<br>Line 145, check the referred figure and should be in result section.	We are not entirely sure what the reviewer is referring to here, but have removed the reference to figure 1 from line 145, as it is already signposted in the Methods above (Line 159).<br><br>We have added a further reference in the Results (Line 236):<br><br>*"After excluding patients who died within the year following discharge (n=22), patients with missing follow-up data (n=20), and patients who could not be benchmarked (n=20), 611 patients from the OMSC group were included in the final benchmark analysis (mean age = 50.6 years, SD = 16.1). See **Figure 1** for details of benchmarking process."*
	2.15	Methods<br>Ethical clearance details should be included at the end of the Methods section (mention institutional approval and reference number if available).	Please see response to issue 1.6 above
	2.16	Results<br>It needs to be clarified in the Results section why 295 patients were not contacted (non-response, loss to follow-up, refusal, etc.).	We have now provided further detail at the beginning of the Results section (and accordingly made the denominator of the percentages consistent). The paragraph now reads as follows (Lines 210–215):<br><br>"*Six hundred and seventy three patients who smoked were admitted to hospital and assessed by a TDA between 1st July 2020 and 30th June 2021. Forty-four percent (n=299) were successfully contacted at six months after discharge from hospital; 104 (15.5%) reported being a non-smoker and 195 (29%) reported still smoking. A further 37 patients (5.5%) had opted out of the intervention prior to the six month mark, and 295 (43.8%) were lost to follow-up (i.e. did not answer the phone or provide a working phone number)."*
	2.17	Results	We thank the reviewer for this good idea, and have now combined the figures as suggested.

Reviewer	Number	Issue	Response/Action
		Consider combining Figures 2 and 3 into a single comparative bar chart for easier visualisation and clearer comparison.	
	2.18	Results<br>Use some Model for the significance results in Table 2 , (p-value and CI)	We have carefully considered your suggestion. However, we elected not to include p-values as fundamentally this is an exploratory, single-site study which does not test any specific predefined hypothesis, and methodological guidelines advise against the unwarranted use of p-values (Wasserstein & Lazar, 2016)<br><br>Likewise we elected not to include confidence intervals as we make no claims about inference to any wider population, nor any true unknown population parameter. Other studies similar to ours, such as the economic evaluation of the CURE project in Manchester, England, have also taken a parsimonious approach and elected not to include any inferential statistics (Evison et al., 2021)<br><br>Furthermore, given the skewed and zero-inflated nature of the cost data in our sample, generating valid inferential statistics would require a method such as non-parametric bootstrapping, which could invite interpretation beyond that which we set out to achieve.<br><br>We would therefore prefer to guard against implying unwarranted precision or generalisability and we hope this is acceptable to the reviewer<br><br>Evison M, Cox J, Howle F, Groom K, Moore R, Clegg H, et al. Health economic analysis for the 'CURE Project' pilot: a hospital-based tobacco dependency treatment service in Greater Manchester. BMJ Open Respir Res [Internet]. 2021 Dec 22;8(1):e001105. Available from: https://bmjopenrespres.bmj.com/lookup/doi/10.1136/bmjresp-2021-001105<br><br>Wasserstein, R. L., & Lazar, N. A. (2016). The ASA Statement on p-Values: Context, Process, and Purpose. The American Statistician, 70(2), 129–133. https://doi.org/10.1080/00031305.2016.1154108
	2.19	Discussion<br>In line 232, check the grammar mistake.	Thank you for spotting this, this has now been corrected (Line 298)

Reviewer	Number	Issue	Response/Action
	2.20	Discussion (Strengths and Limitations)<br>Regarding the societal cost, you can mention from previous literature.	We have now added examples and references to the relevant sentence in the limitations sections. The sentence now reads as follows:<br><br>*"In terms of limitations, the estimated costs do not include post-discharge costs such as local authority Stop Smoking Services, to which some patients were referred, nor do they do not include wider societal costs such as lost productivity from being in hospital or higher pension costs due to increased lifespan (34,35)"* (Lines 334–337)
	2.21	Discussion (Strengths and Limitations)<br>As you mentioned in the limitation that smoking status was not biochemically verified, it means self-report may overestimate quit rates. In discussion section, you can mention how the cost per quit changes if fewer people actually quit than reporting. Additionally, You can add whether results might differ in Non-Urban or post pandemic settings.	This is a valid concern. We agree that reliance on self-reported abstinence may lead to some overestimation of quit rates. In our study, follow-up at six months was conducted by telephone by the Tobacco Dependence Advisors as part of routine care, and carbon monoxide verification was neither feasible or part of standard practice. This reflects the practical realities of treatment initiated during hospital admission, where follow-up commonly occurs after discharge and patient contact is often remote.<br><br>However, we have endeavoured to estimate a 'worst-case scenario' cost per quit and ICER, incorporating a 40% reduced quit rate based on an assumption that CO-validated quit rates would be lower than self-reported quit rates (and incorporating an elevated background quit rate, in response to a similar concern from reviewer 1). This 40% reduction was estimated on the basis of findings from the CURE project pilot, which was conducted in a similar urban UK setting (Evison et al, 2020). These calculations are outlined in detail in a new section of the supplementary material (see section S4), and have been included in the main manuscript Methods (Lines 129-132), Results (Lines 227-231), and Discussion (Lines 291-294).<br><br>We have also now included an additional sentence in the limitations section of the Discussion regarding potential differences in other settings (Lines 346-347).<br><br>Evison M, Pearse C, Howle F, Baugh M, Huddart H, Ashton E, et al. Feasibility, uptake and impact of a hospital-wide tobacco addiction treatment pathway: Results from the CURE project pilot. Clin Med (Northfield Il) . 2020 Mar 18;20(2):196–202.

VERSION 2 - REVIEW

**Reviewer**	**1**
**Name**	**Gawde, Nilesh**
**Affiliation**	**Tata Institute of Social Sciences**
**Date**	**10-Nov-2025**
**COI**	

Thanks for addressing the comments. There are no pending comments.

**Reviewer**	**2**
**Name**	**Goel, Sonu**
**Affiliation**	**PGIMER, SCHOOL OF PUBLIC HEALTH**
**Date**	**29-Oct-2025**
**COI**	

Thank you for submitting the revised version of your manuscript. The authors have addressed the previous comments thoughtfully, and the manuscript has improved considerably in clarity and presentation.
